# A rare case report: An early second trimester cystic hygroma with hydrops fetalis

**DOI:** 10.1016/j.radcr.2024.10.065

**Published:** 2024-11-11

**Authors:** Mesfin Ayalew Tsegaye, Alemayehu Nigusssie Adugna, Abel Benti Abchale, Solomon Muleta Ayano, Rebecca Haile Tesfay

**Affiliations:** Department of Obstetrics and Gynecology, Dilla University, College of Medicine and Health Sciences, Dilla, Ethiopia

**Keywords:** Cystic hygroma, Hydrops fetalis, Nonimmune, Effusion, Aneuploidy

## Abstract

Cystic hygroma is a congenital lymphatic malformation characterized by fluid-filled cysts, often located in the neck or axillary regions, and is associated with significant fetal morbidity and mortality. This case report details a 32-year-old gravida 7 para 6 at 15 weeks of gestation, diagnosed prenatally with a large cystic hygroma exhibiting septation in the cervicoccipital and axillary region, accompanied by bilateral pleural effusion, pericardial effusion and subcutaneous edema. This report underscores the importance of early diagnosis and the need to carefully consider management options in cases of cystic hygroma with fetal hydrops.

## Introduction

Cystic hygromas are congenital lymphatic malformations characterized by fluid-filled cysts resulting from lymphatic drainage obstruction, typically at the lymphatic-venous junction [1]. Their incidence ranges from 1 in 6000 to 1 in 16,000 live births and is associated with significant perinatal morbidity and mortality, particularly when accompanied by hydrops fetalis [2]. These malformations predominantly occur in the neck region (75%), with a smaller percentage found in the axillary area (10%-20%), mediastinum and groin [1,3]. Cystic hygromas are often linked to chromosomal abnormalities, most notably Turner syndrome, and can present alongside structural anomalies such as fetal hydrops [2,4]. Hydrops fetalis, defined as excessive fluid accumulation in fetal body cavities, is primarily nonimmune and accounts for 95% of cases. Approximately 46% of nonimmune hydrops cases are idiopathic, while known causes include cardiovascular disorders and infections [5]. The prognosis for fetuses with cystic hygromas and hydrops is poor, with mortality rates nearing 100% when hydrops is present [3,4]. However, advancements in prenatal management, including intrauterine sclerotherapy with agents like bleomycin or OK-432, have shown promise in cases without chromosomal abnormalities [3,^6^,7]. Early diagnosis and intervention are crucial in managing these complex cases to improve outcomes in resource-limited settings.

## Case presentation

This is a 32-year-old gravida 7 para 6 mother who does not recall her last normal menstrual period but reports being amenorrheic for the past 4 months. She has been married for 14 years. Her firstborn child passed away at the age of 2, 14 years ago. The rest of her children are currently aged between 12 years and 2 years. She presented with a referral for an obstetric ultrasound examination.

The patient has no history of surgeries or major illnesses. She has not experienced any abortions or deliveries of infants with congenital anomalies. She is Rh positive, and her husband is not a relative. There is no history of exposure to teratogenic substances. She has never smoked and denies any alcohol consumption during this pregnancy. Additionally, she has not made any significant dietary changes; her routine diet consists primarily of a local vegetarian diet rich in green leafy vegetables.

A transabdominal ultrasound of the gravid uterus was performed, revealing a single live intrauterine fetus with a gestational age of 15 weeks. The cervicoccipital region was surrounded by a multiseptated cystic mass. Color Doppler imaging did not show any blood flow within the lesion. Bilateral axillary cystic masses were also noted, with no Doppler flow detected. No skull defects were observed. Additionally, there was evidence of pericardial and pleural effusion, along with generalized skin edema. No other significant finding was present.

**Fig. 1:**
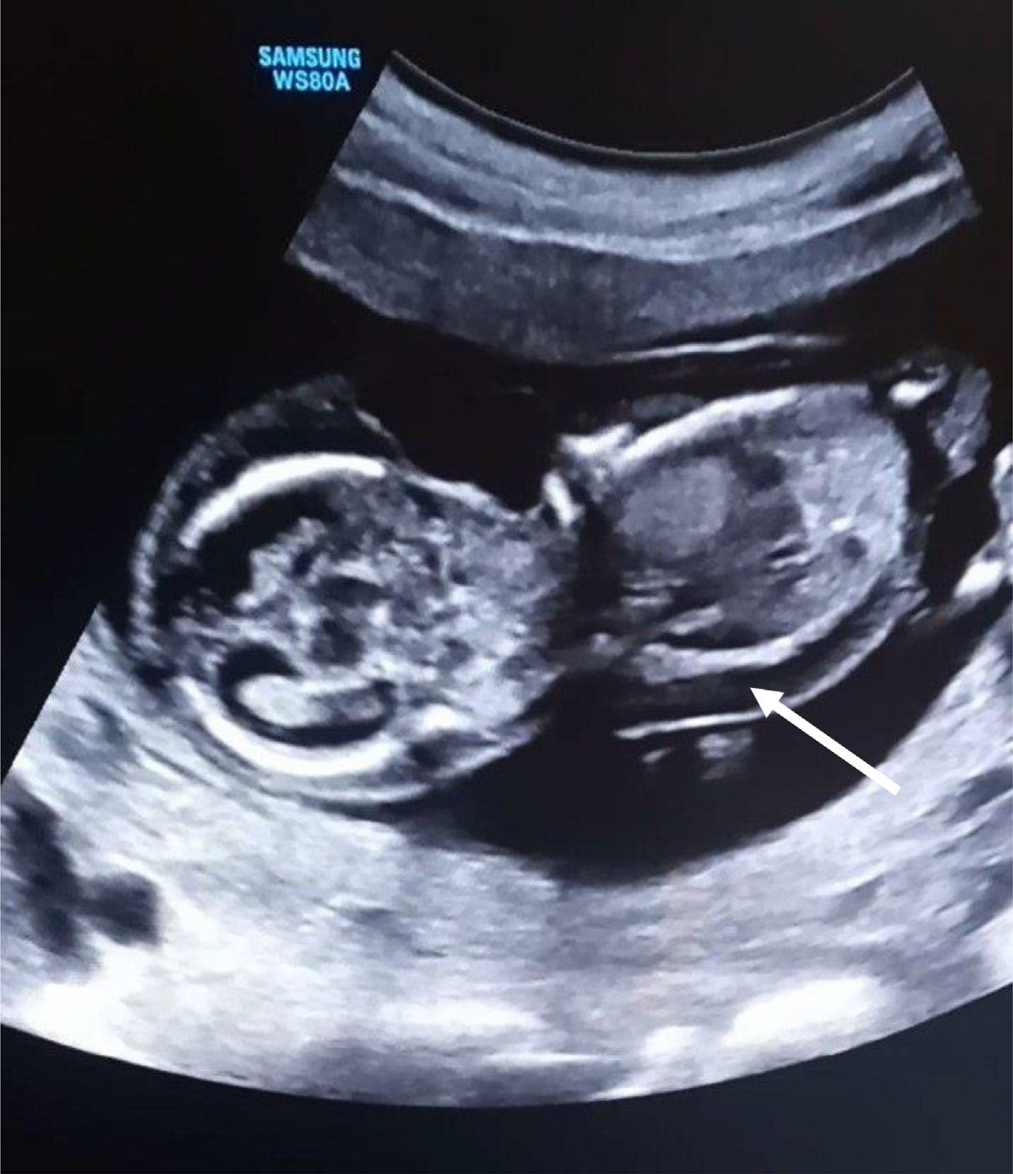
diffuse subcutaneous edema (white arrow).

**Fig. 2:**
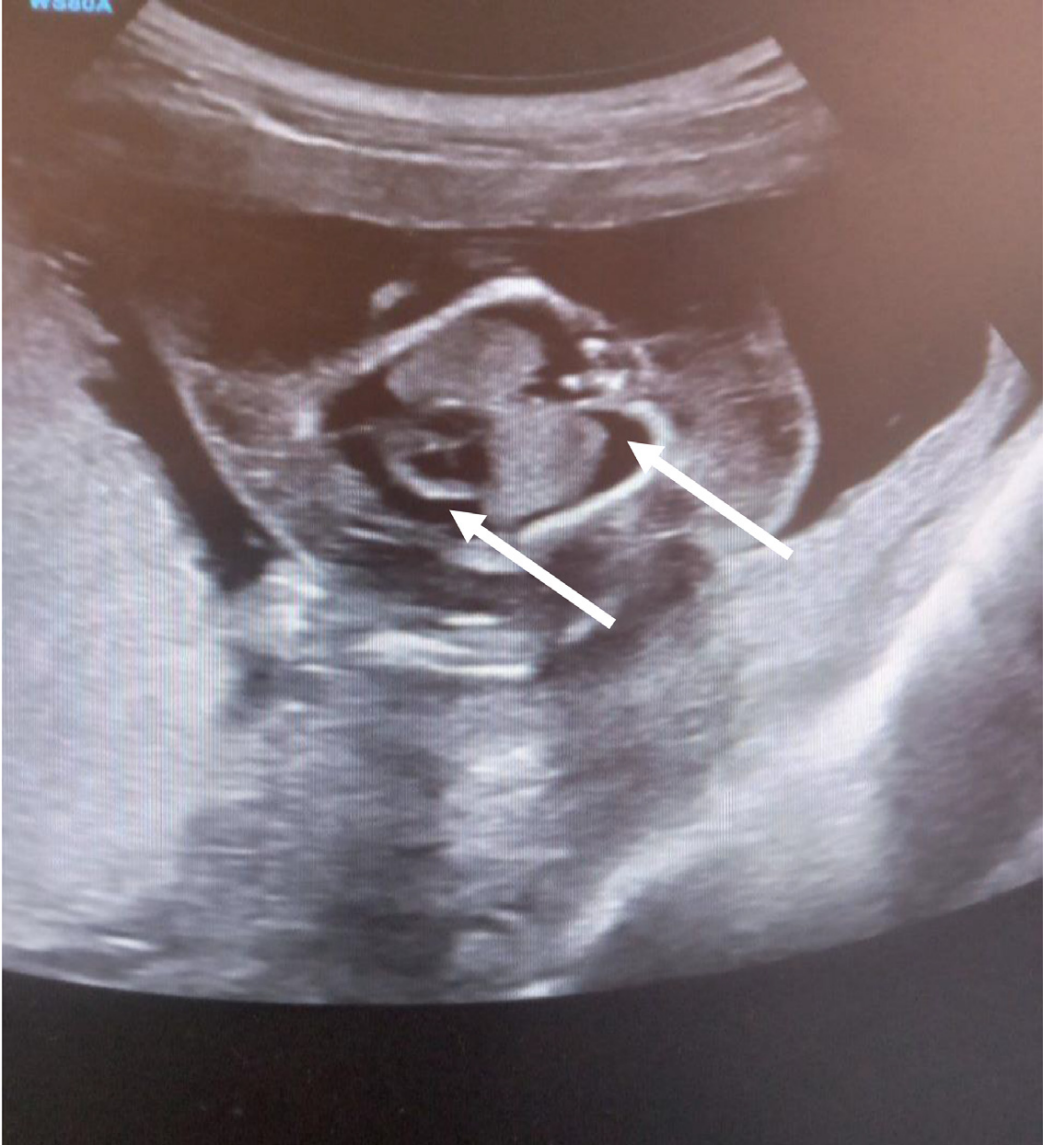
bilateral pleural effusion and pericardial effusion (white arrows).

**Fig. 3:**
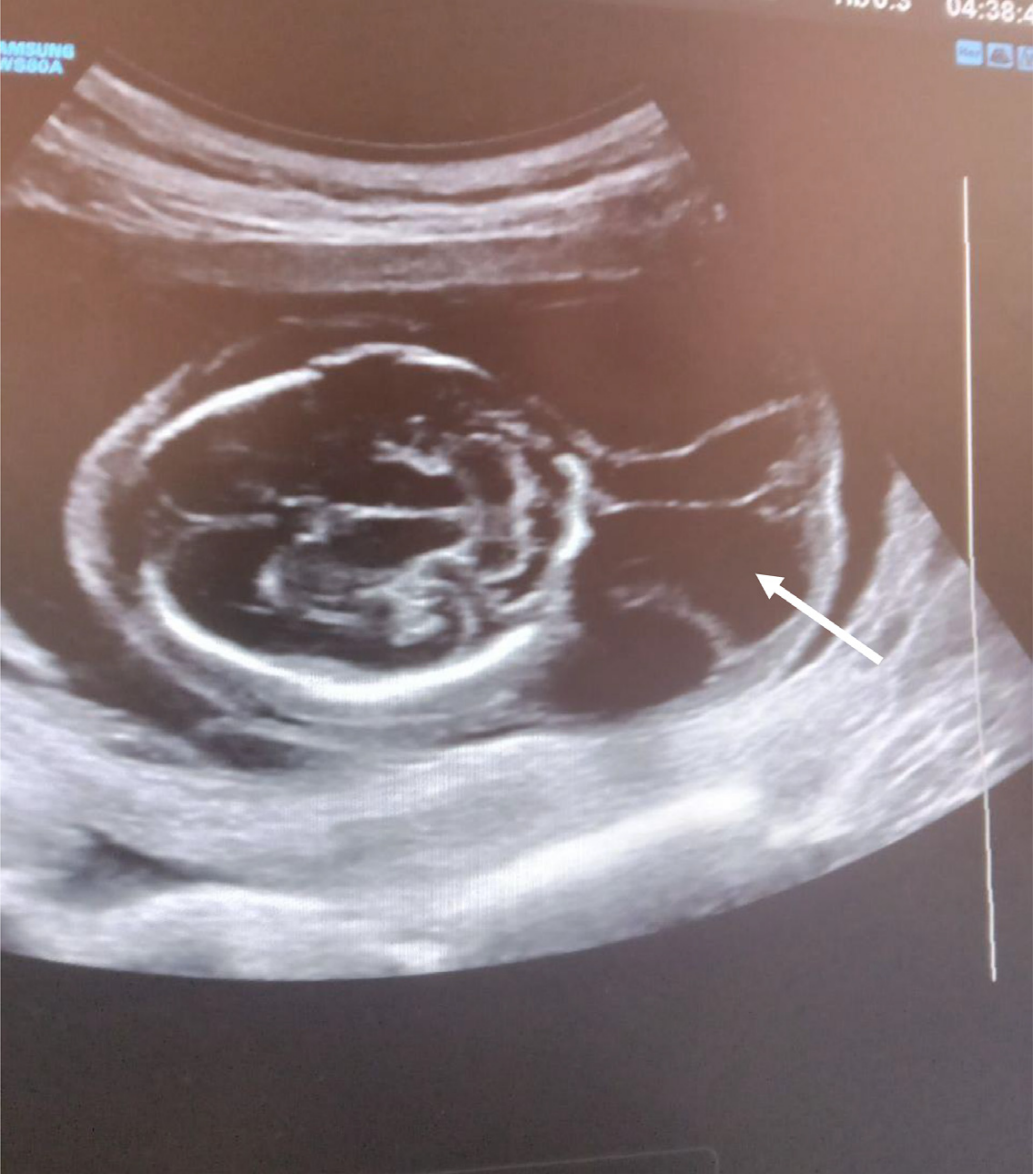
Multiseptated cervicoccipital cyst with no color Doppler flow (white arrow).

**Fig. 4:**
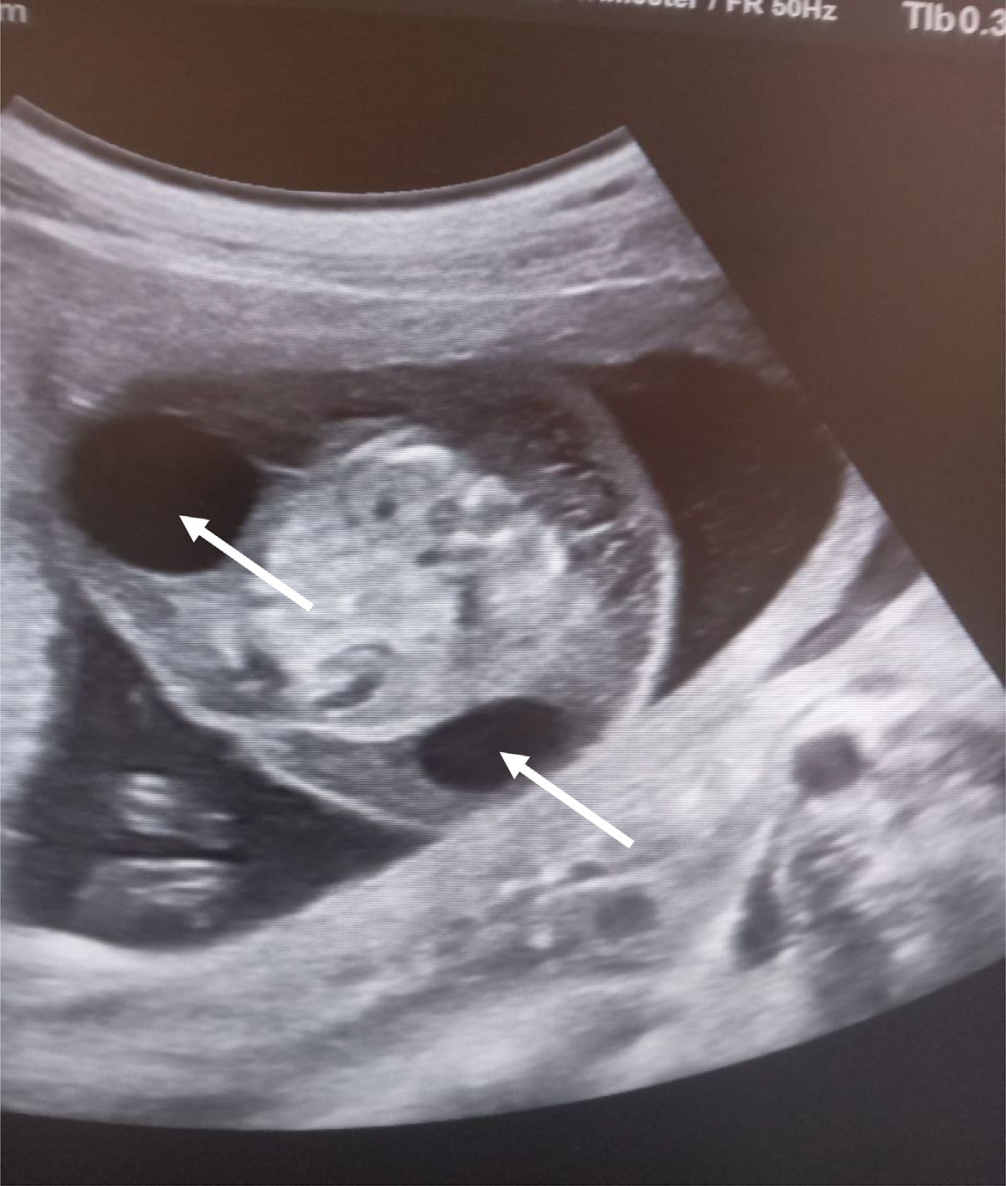
bilateral axillary cyst (white arrows).

**Fig. 5:**
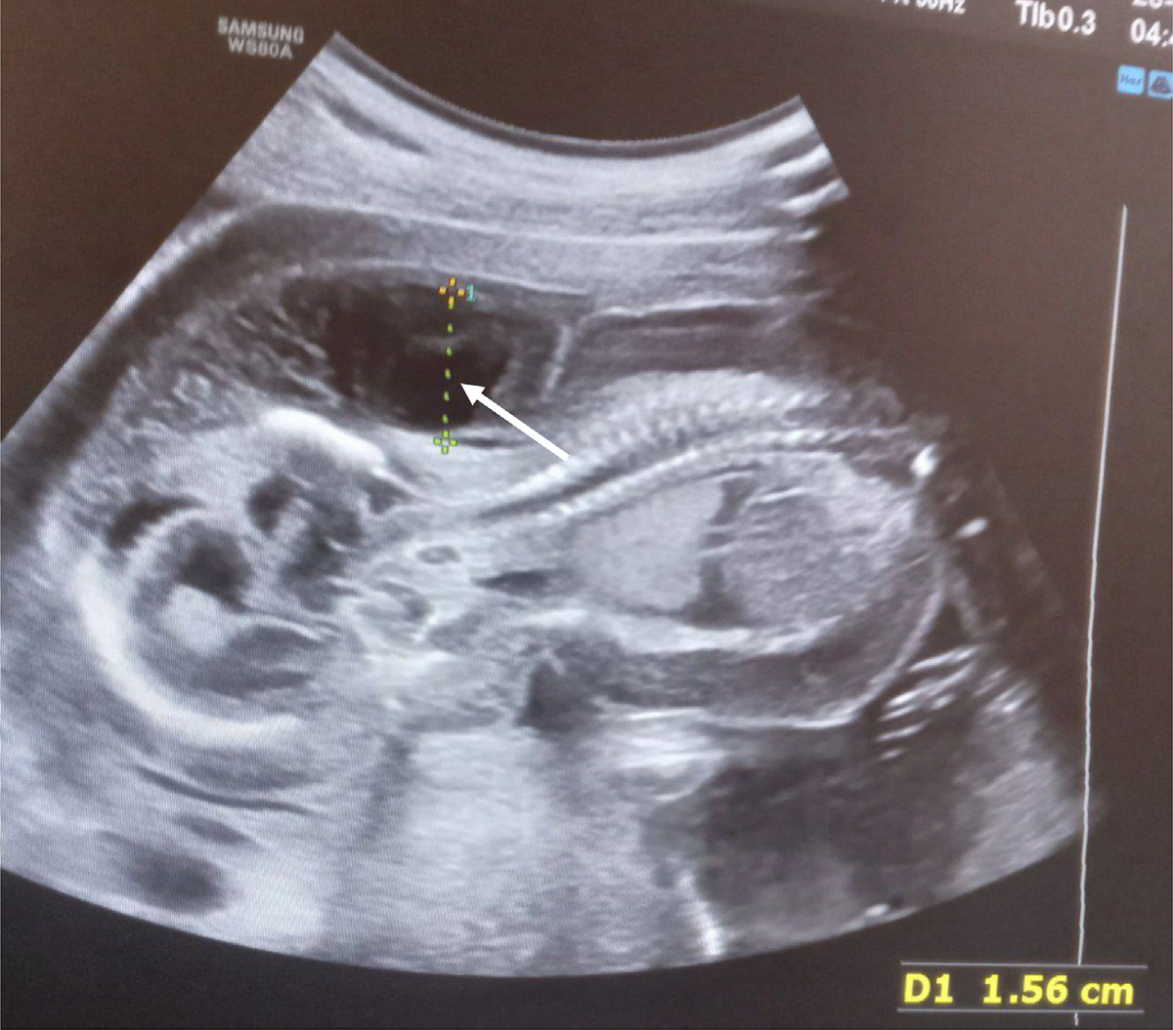
nuchal thickness of 15.6 mm (white arrow).

**Fig. 6:**
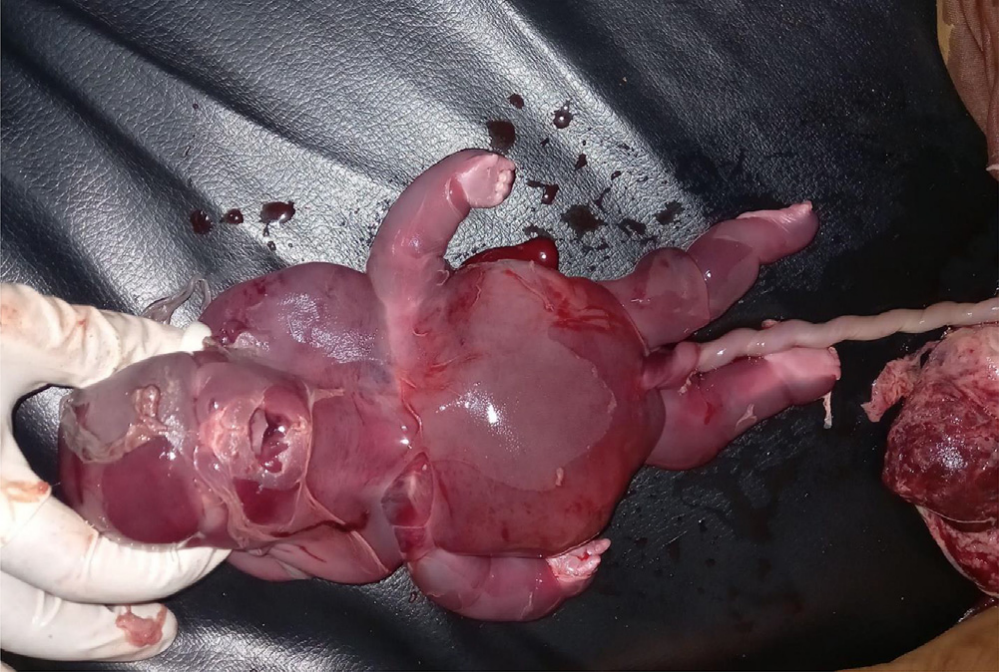
Aborted fetus with cervicoccipital swelling and overall hydrops.

Parents rejected genetic testing before and after abortion, so it was not conducted. They were counseled on the prognosis of the diagnoses and given the choice of termination of the pregnancy, although they denied termination of the pregnancy due to their religious values. Upon subsequent follow-ups, worsening of the subcutaneous edema and pleural effusion was noted. Finally 3 weeks after the diagnosis was made, the mother appeared to the emergency department with vaginal bleeding and lower abdominal pain, which ended in the spontaneous abortion of the fetus (Fig. 1, Fig. 2, Fig. 3, Fig. 4, Fig. 5, Fig. 6).

## Discussion

This case involves cystic hygroma accompanied by hydrops fetalis. Cystic hygromas are believed to arise from remnants of embryonic lymphatic tissue, which retains the ability to proliferate [8]. They can be diagnosed as early as 10 weeks of gestation and have multifactorial causes, with genetic aberrations being the primary factor. Approximately 50% to 80% of cystic hygromas are associated with aneuploidy, such as Turner syndrome and trisomy 21 [2,4]. Cystic hygroma can coexist with hydrops fetalis; Chervenak et al. found this association in 13 out of 15 fetuses [9]. The presence of cystic hygroma alone is linked to a poor prognosis, especially when accompanied by adverse factors such as septation, size greater than 6 mm, and fetal hydrops [2]. Some cystic hygromas may resolve if there are no chromosomal abnormalities and alternate lymphatic channels open [[2], [3], [4]]. Nonimmune fetal hydrops occurs in approximately 1 in 1,700 to 1 in 3,000 pregnancies. Depending on severity, it may present with generalized edema (anasarca), placentomegaly, ascites, and pleural or pericardial effusion. Various causes contribute to nonimmune hydrops, with 46% being idiopathic. The remaining cases are attributed to cardiovascular disorders, chromosomal anomalies, lymphatic abnormalities, metabolic disorders, and fetal tumors. Prognosis varies, with some causes having a favorable outcome, while structural defects, chromosomal abnormalities, or genetic metabolic disorders typically indicate a poor prognosis [5]. Chronic hypoxia due to compression of thoracic structures is a leading cause of fetal demise [3]. Treatment options such as intralesional bleomycin or OK 432, along with fluid removal, have shown effectiveness in cases without chromosomal abnormalities [3,^6^,7]. However, for cystic hygromas associated with fetal hydrops, where mortality is close to 100%, early termination of pregnancy is often recommended as the preferred management approach [3].

## Conclusion

A detailed ultrasound evaluation is crucial for the early detection and management of cystic hygroma with fetal hydrops. Early termination of the pregnancy should also be considered due to the high mortality rate of the fetus.

## Patient consent

Written and informed consent for publication of their case was obtained from the patient.
